# Backward Walking Training Impacts Positive Effect on Improving Walking Capacity after Stroke: A Meta-Analysis

**DOI:** 10.3390/ijerph19063370

**Published:** 2022-03-12

**Authors:** Hongwei Wen, Min Wang

**Affiliations:** Department of Physical Education, Shanghai University of Finance & Economics, No. 777 Guoding Road, Yangpu District, Shanghai 200433, China; mswhw@163.com

**Keywords:** backward walking training, walk function, stroke, meta-analysis

## Abstract

Objective: The meta-analysis aimed to investigate the potential effect of backward walking training (BWT) on walking function improvement among stroke patients. Data sources: Eligible studies were systematically searched in PubMed, Embase, Web of Science, and Cochrane Library. Methods: Heterogeneity among enrolled studies was assessed. Weighted mean difference (WMD) with its 95% confidence interval (CI) was used to pool the outcomes. Results: Seven articles were included. BWT significantly improved motor functions of stroke patients including 10-meter walk test (WMD (95% CI) = 0.11 (0.01, 0.21) meters/second; *p* = 0.03); cadence (WMD (95% CI) = 4.00 (0.99, 7.02) step/minute; *p* < 0.01); Berg balance scale (WMD (95% CI) = 4.38 (2.60, 6.15); *p* < 0.01); paretic step length (WMD (95% CI) = 5.32 (1.97, 8.67) cm; *p* < 0.01); and stride length (WMD (95% CI) = 6.61 (0.70, 12.51) cm; *p* = 0.03) as compared with control group. Conclusion: Our study revealed that BWT had a positive influence on walking function improvement among patients after stroke.

## 1. Introduction

Walking function limitation and motor control impairments are the most common problems among subjects after stroke [1], which leads to reduced quality of life. According to previous data, up to 80% of the poststroke population are affected by gait dysfunction [2]. Notably, independent walking ability of stroke patients would be more likely reestablished if stroke patients could sooner attain the ability to ambulate [3,4]. Therefore, it would be a major goal for stroke patients to improve this modifiable risk factor.

Multiple therapeutic approaches have been developed to improve walking function among stroke survivors, such as lower-extremity strengthening [5] and virtual reality exercises [6]. While, ambulatory deficits still remain as a persistent problem for the poststroke population. More recently, physical activity interventions play roles in reducing the risk of stroke and decreasing other risks associated with cardiovascular diseases, including hypertension and obesity [7]. Walking is accessible, low cost, and the most popular form of exercise around the world [8]. Thus, various types of aerobic exercises have been put forward. More recently, backward walking training (BWT) has been focused on among stroke patients, which is recognized as a potential tool to improve mobility function after stroke through enhancing balance and self-efficacy [9,10,11].

Several studies have been designed to explore the effect of BWT on the walking function of stroke patients. However, the potential role of BWT in walking functional recovery appears to be controversial [11,12,13]. For example, Wang et al. demonstrated that BWT was beneficial for balance performance among patients with a high risk of fall [13]. Moreover, BWT has been recommended as a supplemented tool along with conventional training in improving walking problems among stroke patients [11]. However, in the study by Kim et al., BWT was not recommended as the best treatment strategy for humans after stroke as compared with conventional treatment [14]. A previous meta-analysis reported that BWT can improve the Berg balance scale (BBS), walk test performance, and gait velocity, but the evidence was relatively low [15].

To further confirm the effect of BWT on the walking function improvement after stroke, we conducted a meta-analysis for randomized controlled trials (RCTs) researching the topics by systematically searching in PubMed, Embase, Web of Science, and Cochrane Library. WMD with its 95% CI was used to evaluate the outcomes, including 10-meter walk test (10MWT), stride length, gait cycle, cadence, BBS, paretic step length, paretic single support, total double support, and paretic step time.

## 2. Methods

### 2.1. Selection Strategy

The meta-analysis was performed based on the Preferred Reporting Items for Systematic Reviews and Meta-analyses (PRISMA) [16].

The eligible studies were thoroughly searched from databases, including PubMed, Embase, Web of Science, and Cochrane Library, until 19 August 2021. The combination of the following search terms was used: “backward”, “walking”, and “stroke” (Supplementary Table S1), and the included studies were selected without language limitation. Moreover, print-out literatures were also searched by manual retrieval, and the references were further checked to explore all relevant papers.

### 2.2. Study Selection

RCTs were included in the present meta-analysis if (1) the subjects were stroke patients; (2) the patients in the experiment group took part in BWT or backward walking treadmill training, and the patients in the control group underwent conventional training or forward walking training; and (3) the study outcome included one or more of the following factors: 10MWT, stride length, gait cycle, BBS, paretic step length, paretic single support, total double support, and paretic step time. Moreover, the data after intervention were also reported.

The exclusion criteria were as follows: (1) the study did not include any outcomes or data after intervention; (2) reviews, comments, and letters; and (3) the study with more reliable information would be included if duplicated data occurred.

### 2.3. Data Extraction and Quality Assessment

The data from pre-designed standardized form were extracted by two investigators independently, and the following information was extracted: the name of the first author, year of publication, study area, age of participants, affected side, course of disease, sex distribution, sample size, intervention plan, intervention cycle, and outcomes. After both of them completed the above data extraction work, they exchanged the audit extraction form.

The Cochrane Collaboration’s tool for assessing risk was selected to assess the quality of included studies [17]. If disagreements occurred during data extraction and quality assessment, it was be solved by discussing with the third investigator.

### 2.4. Statistical Analysis

In order to investigate the possible role of BWT on stroke, weighted mean difference (WMD) with its 95% CI was used to pool the outcome.

Cochran’s Q test and I^2^ test were used to assess heterogeneity among enrolled studies. Studies with *p* < 0.05 and/or I^2^ > 50% were defined as significant heterogeneity occurring among included studies; otherwise, the heterogeneity was not significant.

Publication bias of included studies was assessed by funnel chart. All statistical analyses were conducted using RevMan5.3.

## 3. Results

### 3.1. Literature Search

**Figure 1:**
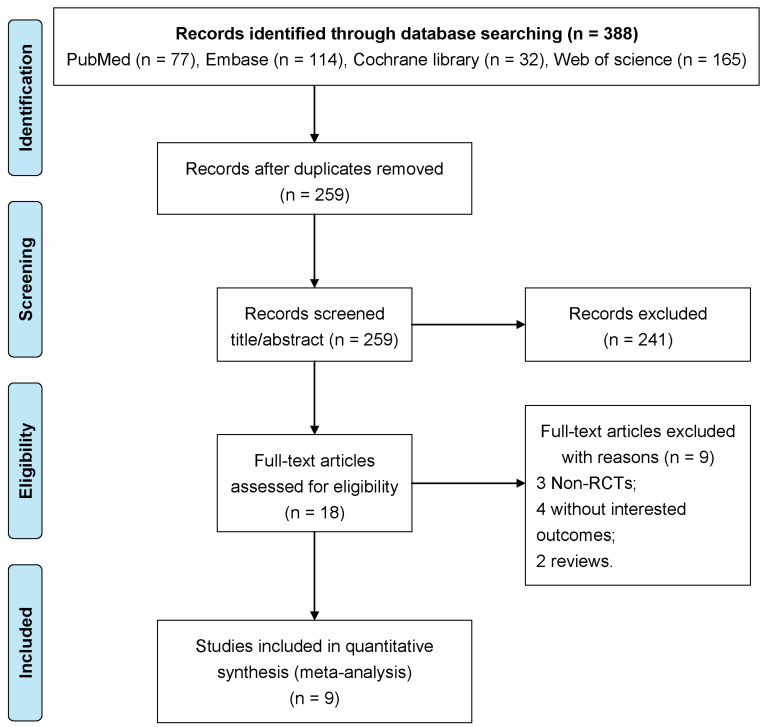
The detailed flow chart for study selection.

The process of literature selection is shown in Figure 1. In this literature search, 77, 114, 32, and 165 articles were retrieved in the PubMed, Embase, Cochrane Library databases, and Web of Science, respectively. In total, 259 articles remained after removing 129 duplicate literatures. After browsing the titles and abstracts, 241 articles were eliminated. Of the remaining 18 articles, 9 articles were deleted by reading the full text. Manual searches failed to obtain studies that could be included in the analysis. Finally, nine articles were included in this meta-analysis [11,12,14,18,19,20,21,22,23].

### 3.2. Characteristics of the Enrolled Studies

As shown in Table 1 and Table 2, the included studies were published ranging from 2005 to 2021, and the studies were conducted in the United States, Japan, Italy, South Korea, and China, respectively. A total of 225 subjects were included, of which 105 cases belonged to BWT group, and 120 patients belonged to the control group. Notably, the study conducted by Takami et al. [20] performed conventional walking training and forward walking treadmill training. Therefore, conventional walking training was used as the control in six studies, and forward walking training was used as the control in four studies (Table 1). Except for the study conducted by Munari et al. [23], where the BWT training time was 40 min, the training time of BWT in other studies was all 30 min, but several studies combined conventional exercise intervention and BWT. Conventional walking training included standing balance training, overground walk training, strengthening, function and mobility activities, gait training, and so on. Moreover, the specific intervention plan and follow-up period of the study were also different in each study.

Except for the significant differences in the age in the study by Rose et al. [19], no significant difference was found in gender composition, age, course, disease side, etc., in other studies (BWT vs. control group, *p* > 0.05) (Table 2).

**Figure 2:**
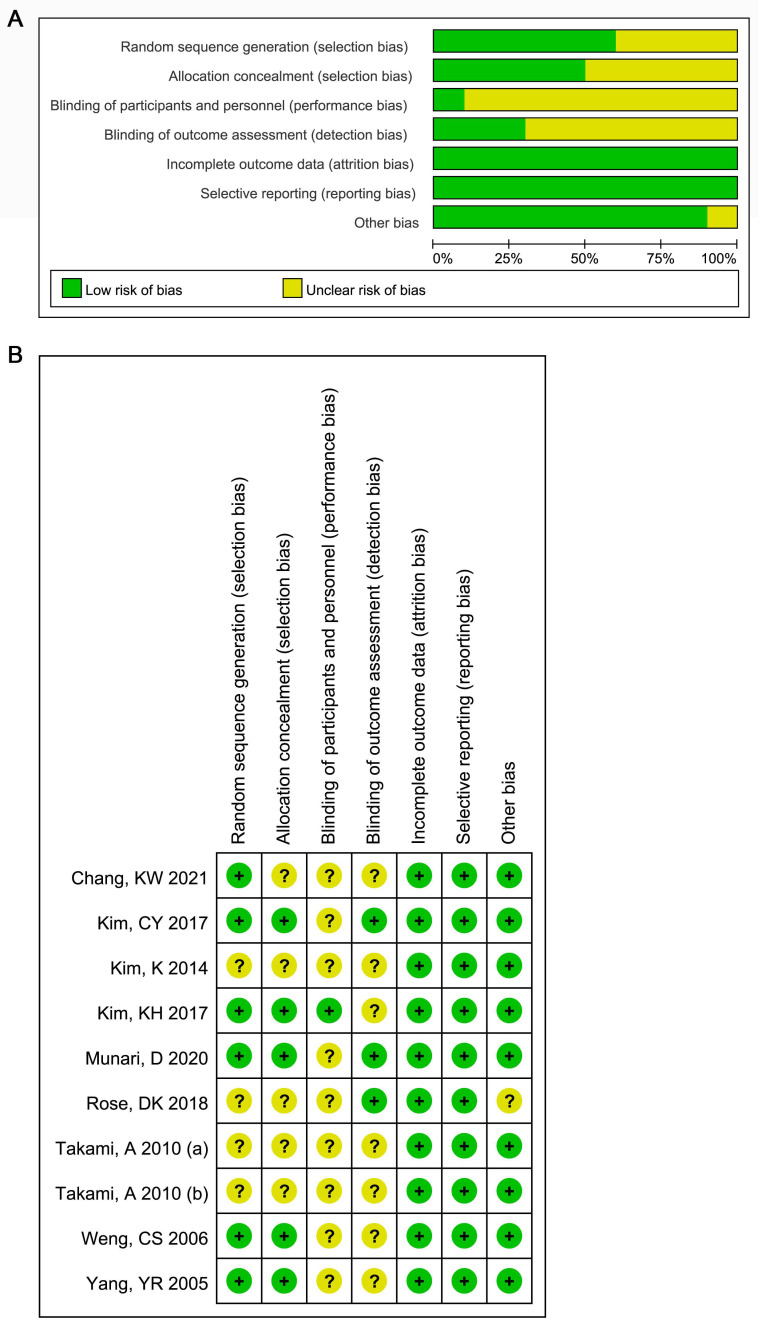
The quality of included studies evaluated by the Cochrane Collaboration’s tool for assessing risk. (**A**) Risk of bias graph; (**B**) risk of bias summary. “+” indicated “Low risk” and “?” indicated “Unclear”.

Since most studies did not clearly report whether blind measurement was designed in the sports rehabilitation experts, stroke patients, and outcome, performance bias and detection bias were thus evaluated as “Unclear”. Four studies did not describe the specific methods of random grouping and allocation concealment, so the selection bias was evaluated as “Unclear” [18,19,20,22]. There is a significant difference in the age of the research subjects in the study by Rose et al. [18], so other bias was defined as “Unclear”. The remaining evaluation items were all “Low risk”. Overall, the methodological bias of the included literature was moderate (Figure 2).

### 3.3. Results of Meta-Analysis

**Figure 3:**
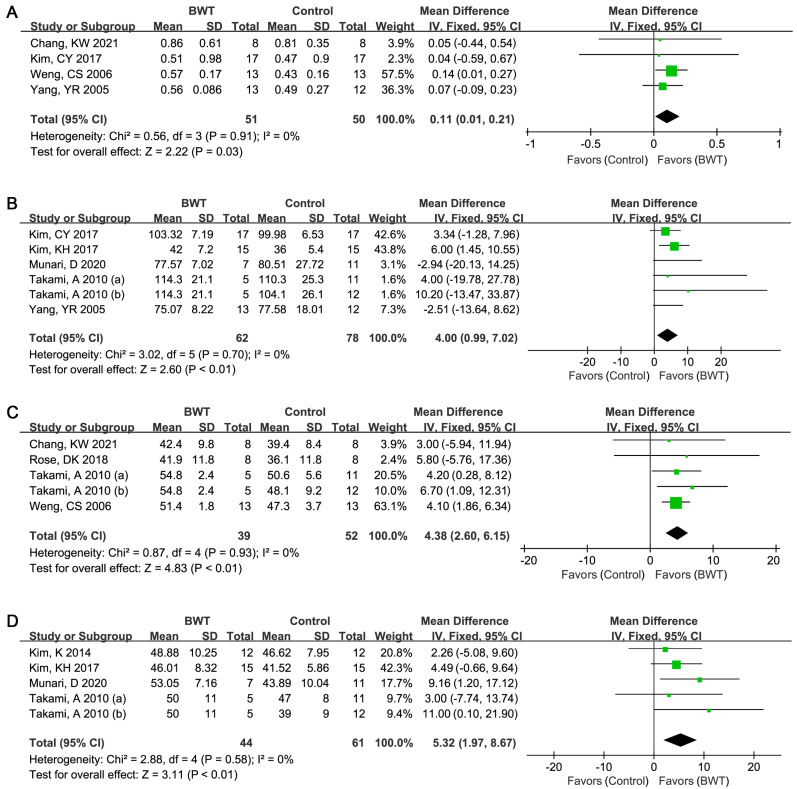
Forest plot for meta-analyzing the role of backward walking training and conventional treatment on 10-Meter Walk Test (10MWT), cadence, Berg balance scale (BBS), and paretic step length. (**A**): 10MWT; (**B**): cadence; (**C**): BBS; (**D**): paretic step length.

Figure 3A–D shows the difference in 10MWT, cadence, BBS, and paretic step length between BWT and control group. Four literatures reported the difference of 10MWT between BWT and control group [11,14,21,22]. There was no significant heterogeneity between the studies (I^2^ = 0%, *p* = 0.91). Figure 3A shows significant improvement of BWT on 10MWT (WMD (95%CI) = 0.11 (0.01, 0.21) meters/second, *p* = 0.03).

In total, five studies reported the evaluation of cadence [11,12,14,20,23]. Figure 3B showed that no significant heterogeneity was observed (I^2^ = 0%, *p* = 0.70), and significant improvement of BWT was found on cadence based on the fixed effect model (WMD (95% CI) = 4.00 (0.99, 7.02) step/minute, *p* < 0.01).

Similarly, no significant heterogeneity was observed between studies focusing on BBS comparison (I^2^ = 0%, *p* > 0.05) [19,20,21,22], paretic step length (I^2^ = 0%, *p* > 0.05) [12,18,20,23]. The combined results based on the fixed effect models showed significant improvement on BBS (WMD (95% CI) = 4.38 (2.60, 6.15), *p* < 0.01, Figure 3C) and paretic step length (WMD (95% CI) = 5.32 (1.97, 8.67) cm, *p* < 0.01, Figure 3D) after BWT.

**Figure 4:**
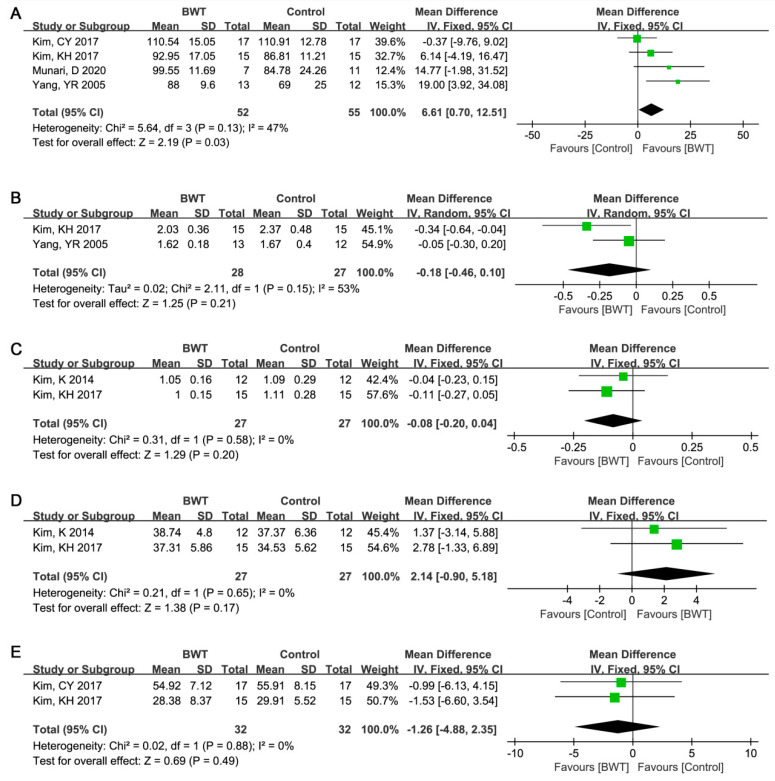
Forest plot for meta-analyzing the role of backward walking training and conventional treatment on stride length, gait cycle, paretic step time, paretic single support, and total double support. (**A**) Stride length; (**B**) gait cycle; (**C**) paretic step time; (**D**) paretic single support; and (**E**) total double support.

The evaluation of stride length, gait cycle, paretic step time, paretic single support, and total double support were shown in Figure 4A–E. There was no significant heterogeneity between studies [11,12,14,23] on stride length (I^2^ = 47%, *p* = 0.13). The combined results based on the fixed-effect models showed significant improvement of BWT on stride length (WMD (95%CI) = 6.61 (0.70, 12.51) cm, *p* = 0.03, Figure 4A). Significant heterogeneity occurred between studies reaching on gait cycle [11,12] (I^2^ > 50%). Based on the random effect model, no significant difference was found between BWT and control group on gait cycle (WMD (95%CI) = −0.18 (−0.46, 0.10), *p* = 0.21, Figure 4B). According to pooled data on the comparison of paretic step time [12,18], paretic single support [12,18], and total double support [12,14], no significant heterogeneity was calculated between studies (I^2^ = 0%, *p* > 0.05). Then, the fixed effect model was used to pool data, and the results showed no significant difference was found between BWT and control group on paretic step time (WMD (95%CI) = −0.08 (−0.20, 0.04) s, *p* = 0.20, Figure 4C), paretic single support (WMD (95%CI) = 2.14 (−0.90, 5.18)%, *p* = 0.17, Figure 4D), total double support (WMD (95%CI) = −1.26 (−4.88, 2.35)%, *p* = 0.49, Figure 4E).

### 3.4. Publication Bias

Due to the small number of included literatures, the number of studies included in each outcome was less than 10. As a result, not only qualitative (funnel chart) but also quantitative test methods (such as Egger test) have relatively low test efficiency. Thus, publication bias was not performed in this meta-analysis.

## 4. Discussion

BWT might offer a number of potential benefits for patients after stroke with long-term disabilities. Based on data from RCTs, the potential effects of BWT on outcomes, including 10MWT, stride length, gait cycle, cadence, BBS, paretic step length, paretic single support, total double support, and paretic step time, were systematically analyzed. Our data showed that BWT significantly improved motor functions of stroke patients, including 10MWT (WMD (95% CI) = 0.11 (0.01, 0.21) meters/second, *p* = 0.03), cadence (WMD (95% CI) = 4.00 (0.99, 7.02) step/minute, *p* < 0.01), BBS (WMD (95% CI) = 4.38 (2.60, 6.15), *p* < 0.01), paretic step length (WMD (95% CI) = 5.32 (1.97, 8.67) cm, *p* < 0.01), and stride length (WMD (95%CI) = 6.61 (0.70, 12.51) cm, *p* = 0.03). No significant difference was found between BWT and control groups in gait cycle, paretic step time, paretic single support, and total double support.

Previous meta-analysis focusing on the effects of aerobic exercise interventions on quality of life demonstrated that aerobic exercise interventions had a significant positive effect on walking speed and endurance [24,25]. A further systematic review showed that, among patients with knee osteoarthritis, backward walking was effective and clinically worthwhile when combined with conventional treatment [26]. Nevertheless, the effects of the backward walking on walking improvement of patients after stroke have not been systematically analyzed. In our meta-analysis, we demonstrated that BWT could significantly improve walking functions, such as 10MWT, cadence, BBS, paretic step length, and stride length. As compared with walking forward, more motor areas of the cortex are activated [27]. When walking backward, visual flow and the absence of peripheral visual feedback would be absent during walking [28]. Then, in order to control the pattern of walking step, backward walking may need a reweighting of sensory feedback [29]. These outcomes, including cadence, BBS, paretic step length, and stride length, were all critical for patients to recover to premorbid environments. Although there was a large difference in the course of stroke among the included subjects, the meta-analysis results showed that the heterogeneity of these outcomes between studies was not significant. Therefore, BWT should be recommended for patients after stroke, and the timing of BWT may be better as early as possible. In addition, according to the characteristics of the subjects, intervention plan, and our meta-analysis results, we suggest that stroke patients with basic walking ability (can be able to walk 10 m or more with or without auxiliary equipment) should take BWT for 30 min every day for 4 weeks and then decide whether to insist on longer training depending on the improvement of walking function.

Meanwhile, our data showed no significant difference was found between BWT and control groups in gait cycle, paretic step time, paretic single support, and total double support. Notably, significant heterogeneity occurred between studies reaching on gait cycle. The heterogeneity may be attributed to difference measurement tool or various backgrounds of included subjects. Then, the conclusion might be valuable for future research. Therefore, the results of the present study should be verified by further studies with larger sample size and longer experiment duration.

There are some strengths in the meta-analysis. Firstly, the statistical heterogeneity of the included literature was small, and studies focusing on most of the outcome indicators had no significant heterogeneity. Secondly, only RCT studies were included in the meta-analysis, which guarantee high methodological quality, small bias, and high credibility of the combined results. Meanwhile, limitations in our meta-analysis should not be ignored. Firstly, the number of included studies and the sample size included in literature were relatively small, and subjects in most researches were Asian. Thus, the extrapolation of meta-analysis results would be limited. Secondly, although the degree of statistical heterogeneity was relatively low, clinical heterogeneity could not be ignored. The intervention plans and follow-up cycles included in the study were different from each other, which might affect the authenticity of the combined results. Thirdly, the long-term effects of BWT could not be evaluated due to the relatively short follow-up time of the included studies. Moreover, this study was unable to compare the effects of BWT and BWT with conventional treatments on the walking function improvement after stroke due to the lack of relevant clinical information. Future research needs to examine the precise dose and recommendation for aerobic exercise, test other exercise modalities, and use larger samples to thoroughly determine long-term exercise effects on mobility in this population.

## 5. Conclusions

In conclusion, BWT significantly improved motor functions of stroke patients, including 10MWT, cadence, BBS, paretic step length, and stride length, and BWT should be recommended for patients after stroke. However, further studies with larger sample size and longer experiment duration should be designed to confirm the present results.

## Figures and Tables

**Table 1:** Interventions included in the study.

Study	Area	Group	Intervention	Follow Up
Chang, KW 2021	China	BWT	30 min conventional walking training + backward treadmill training	3 times/week, 4 weeks
		Control	30 min strengthening, function and mobility activities, gait training	
Kim, CY 2017	Korea	BWT	30 min Backward Walking Training	3 times/week, 3 weeks
		Control	30 min Standing Balance Training	
Kim, K 2014	Korea	BWT	30 min Progressive Body Weight Supported backward walking treadmill training	6 times/week, 6 weeks
		FWT	30 min Progressive Body Weight Supported forward walking treadmill training	
Kim, KH 2017	Korea	BWT	30 min Progressive Body Weight Supported backward walking treadmill training	5 times/week, Four weeks
		FWT	30 min Progressive Body Weight Supported forward walking treadmill training	
Munari, D 2020	Italy	BWT	40 min backward walking treadmill training	3 times/week, 4 weeks
		FWT	40 min forward walking treadmill training	
Rose, DK 2018	USA	BWT	30 min Backward Walking Training	8 sessions during the inpatient period
		Control	30 min standing Balance Training	
Takami, A 2010	Japan	BWT	30 min conventional walking training and 10 min backward treadmill training	6 times/week, 3 weeks
		FWT	30 min conventional walking training and 10 min forward treadmill training	
		Control	40 min overground walk training	
Weng, CS 2006	China	BWT	30 min conventional walking training and 30 min backward walking training	5 times/week, 3 weeks
		Control	60 min conventional walking training	
Yang, YR 2005	China	BWT	30 min backward Walking Training and 40 min conventional training	3 times/week, 3 weeks
		Control	40 min strengthening, function and mobility activities, gait training	

BWT, Backward Walking Training; FWT, Forward walking training.

**Table 2:** Characteristics of nine included studies in this meta-analysis.

Study	Group	N	Sex, M/F	Age, Years	Post Stroke Duration	Affected Side, L/R	Ischemic/ Hemorrhage	Severity of Stroke Patients
Chang, K.W., 2021	BWT	8	6/2	52.39 ± 6.06	22.93 ± 13.7 months	5/3	1/7	Hemiplegia; BMS of lower extremity ≥4; ability to walk at least 11 m; no visual defects or hemianopia
	Control	8	5/3	54.38 ± 14.05	43.64 ± 32.69 months	1/7	5/3	
Kim, C.Y., 2017	BWT	17	7/10	63.83 ± 7.27	7.99 ± 3.58 months	10/7	8/9	Lower-extremity BMS of 3 or 4; ability to walk 14 m; hemiparesis
	Control	17	9/8	63.33 ± 11.60	7.12 ± 2.32 months	8/9	11/6	
Kim, K., 2014	BWT	12	9/3	50.25 ± 16.69	11.83 ± 3.46 months	4/8	NR	No joint contracture, fractures, or hemianopia; functional gait index scores exceeding three points
	FWT	12	8/4	52.75 ± 9.21	11.00 ± 4.22 months	6/6		
Kim, K.H., 2017	BWT	15	11/4	48.27 ± 16.05	10.93 ± 3.67 months	10/5	4/11	No joint contracture, pain, fractures, or hemianopia; FAC scores exceeding four and five points
	FWT	15	7/8	50.73 ± 13.50	11.27 ± 4.10 months	8/7	6/9	
Munari, D., 2020	BWT	7	6/1	58.29 ± 10.14	84 ± 40.8 months	2/5	NR	Ability to walk backward and forward for more than 5 m without a brace or other aid
	FWT	11	7/4	64.73 ± 8.32	84 ± 44.4 months	6/5		
Rose, D.K., 2018	BWT	8	4/4	53.8 ± 12.1	8.5 ± 4.2 days	5/3	NR	Able to maintain upright standing posture with moderate assistance; vision within functional limits
	Control	8	2/6	66.6 ± 7.3 *	7.8 ± 3.3 days	5/3		
Takami, A., 2010	BWT	12	6/6	66.1 ± 6.3	13.2 ± 8.4 days	5/7	7/5	Success walking 10 m using braces or canes; Functional Independence Measure-Locomotion score of 5 or lower
	FWT	12	9/3	71.1 ± 10.6	14.7 ± 8.1 days	7/5	11/1	
	Control	12	5/7	66.9 ± 10.6	13.7 ± 8.9 days	2/10	11/1	
Weng, C.S., 2006	BWT	13	8/5	51 ± 12	62 ± 24 days	6/7	8/5	Lower-extremity BMS of 3 or 4; no joint contracture; ability to walk at least 10 m without assistance or ankle-foot orthosis
	Control	13	9/4	50 ± 14	63 ± 34 days	7/6	6/7	
Yang, Y.R., 2005	BWT	13	10/3	63.38 ± 7.7	5.45 ± 3.03 months	5/8	NR	Hemiplegia; lower-extremity BMS at 3 or 4; ability to walk 11 m with/without a walking aid or orthosis
	Control	12	9/3	63.42 ± 11.06	7.33 ± 2.42 months	4/8		

L, left; R, right; M, male; F, female; NR, not reported; BMS, Brunnstrom motor stage; FAC, Functional Ambulatory Category; BWT, Backward Walking Training; FWT, Forward walking training; *, *p* < 0.05 (BWT vs. control group).

## Data Availability

Data sharing is not applicable to this article as no new data were created or analyzed in this study.

## Supplementary Materials

The following supporting information can be downloaded at: https://www.mdpi.com/article/10.3390/ijerph19063370/s1, Table S1: Literatures selection in PubMed (The retrieval time: 20210819).
