# Supplementary material for: Backward Walking Training Impacts Positive Effect on Improving Walking Capacity after Stroke: A Meta-Analysis
Source: Int J Environ Res Public Health. 2022 Mar 12;19(6):3370. doi: 10.3390/ijerph19063370 (PMC8956083; doi:10.3390/ijerph19063370)
Supplement: Supplementary file 1 [file ijerph-19-03370-s001.zip › ijerph-1570360-supplementary.pdf]

Supplementary Table S1. Literatures selection in PubMed (The retrieval time: 20210819).

Search	Query	Items Found
#1	(Backward[All Fields] AND ("walking"[MeSH Terms] OR “walking” [All Fields]))	1271
#2	("stroke"[MeSH Terms] OR "stroke"[All Fields])	372313
#3	#1 AND #2	77
